# Mood Monitoring in Schools: A Promising Alternative to Single-Time-Point Screening

**DOI:** 10.3390/ijerph23040423

**Published:** 2026-03-27

**Authors:** Shane L. Rogers, Nicole Brown, Kathryn Campbell, Matthew Goulding

**Affiliations:** 1 School of Arts and Humanities, Edith Cowan University, Perth 6000, Australia; 2 Association of Independent Schools of Western Australia, Perth 6000, Australia; 3 Institute of Education and Humanities, University of Wales Trinity Saint David, Lampeter SA48 7ED, UK

**Keywords:** mental health, schools, mood monitoring, screening, emotional well-being, adolescents, classification, thresholds

## Abstract

**Highlights:**

**Public health relevance—How does this work relate to a public health issue?**

- School-based mental health screening is widely used to identify young people who may require early support, yet it typically relies on single-time-point assessments.
- Emotional well-being fluctuates across weeks, raising important public health questions about the stability and accuracy of threshold-based screening decisions.

**Public health significance—Why is this work of significance to public health?**

- Nearly one in five students showed classification changes across repeated assessments, indicating that screening outcomes can depend on when measurement occurs.
- While single-time-point and averaged-score classifications showed high agreement, a conservative stability-based criterion identified substantially fewer students for follow-up.

**Public health implications—What are the key implications or messages for practitioners, policy makers and/or researchers in public health?**

- Repeated mood monitoring provides additional temporal context for interpreting screening classifications and may reduce reliance on single assessment snapshots.
- Public health implementation of school-based screening should carefully consider how repeated data are aggregated, as decision rules directly influence identification outcomes.

**Abstract:**

School-based mental health screening typically relies on single-time-point assessments, which assume that students’ emotional well-being is sufficiently stable for classification based on a single measurement. The present study examined this assumption by evaluating the stability of emotional well-being classifications under repeated mood monitoring. Students from two secondary schools (United Kingdom, *n* = 413; Australia, *n* = 354) completed the Brief Emotional Experience Scale weekly across six to seven weeks. Emotional well-being classifications were examined relative to a predefined low well-being threshold to assess stability across time, and a post-monitoring survey examined students’ self-reported perceptions of the monitoring experience. Most students (78%) showed consistently above-threshold classifications across monitoring occasions, while a small proportion (5%) showed persistently low classifications. However, 17% of students fluctuated above and below the low well-being threshold across weeks, indicating that classification status for this group was sensitive to assessment timing. When monitoring data were aggregated using different decision rules, the proportion of students flagged as low well-being varied substantially, ranging from approximately 5% under a conservative stability-based criterion to around 12% when classifications were based on averaged monitoring scores. Classifications derived from averaged monitoring scores showed high agreement with single-time-point classifications, while identifying a partially different subset of students as low well-being, underscoring the sensitivity of threshold-based decisions to classification approach. Student feedback provided preliminary contextual insight into the acceptability of repeated monitoring under routine school conditions, with over half of respondents reporting that the process supported their emotional understanding. A substantial minority also reported greater inclination to talk with others about their well-being. Overall, the findings indicate that single-time-point screening may provide an incomplete basis for emotional well-being classification for some students, and that repeated assessment offers additional temporal context for interpreting threshold-based screening decisions.

## 1. Introduction

In this article, we examine mood monitoring as an alternative temporal approach to traditional single-time-point mental health screening in schools. Rather than treating emotional well-being as a static construct captured adequately by a single assessment, mood monitoring allows emotional experiences to be tracked over time. This temporal information may be particularly relevant for interpreting screening classifications that rely on threshold-based decisions.

Using data from a cross-national study conducted in two secondary schools, we examined weekly fluctuations in students’ emotional well-being across a school term. The primary aim of the study was to assess the proportion of students whose scores crossed above and below a low well-being threshold over time, thereby evaluating the stability of screening classifications derived from repeated assessment. A secondary aim was to examine students’ perceptions of the personal impact of the monitoring process, with a focus on emotional understanding and communication rather than intervention effects.

Together, these aims address a central assumption of school-based mental health screening: that a single measurement provides a sufficiently representative basis for classification. By examining how emotional well-being classifications change when measured repeatedly across a school term, the study clarifies the limits of single-time-point screening and the potential value of repeated monitoring. At the same time, examining students’ perceptions of the personal impact of mood monitoring provides contextual information that complements classification data, helping to situate observed patterns of emotional change within students’ everyday experiences of tracking their emotions over time.

### 1.1. School-Based Mental Health Screening

Schools are increasingly recognised as central to identifying and supporting young people experiencing mental health difficulties. Globally, around one in five children and adolescents experience a mental health condition at any given time [1,2,3], and emotional problems such as anxiety and depression often emerge during the school years [4,5]. Left unaddressed, these problems can affect academic engagement, peer relationships, and long-term well-being [6,7,8]. In response to growing concern about youth mental health, many education systems have begun incorporating systematic approaches to identify students who may need support, often through school-based mental health screening initiatives [9,10,11].

The most common screening approach involves administering questionnaires on a single occasion (often annually or once per term) to students or teachers [12,13,14,15,16,17,18,19,20,21,22]. These surveys typically ask students to reflect on recent weeks or months and then classify them into well-being categories. Those below a threshold are flagged for follow-up. This model is attractive because it is simple, time-efficient, and relatively low-cost to deliver at scale. Yet it rests on a key assumption: that a snapshot of mood or well-being at one moment provides a sufficiently representative basis for screening classification.

### 1.2. Limitations of Single-Time-Point Screening

Empirical research increasingly challenges the notion that a single-time-point assessment can provide an accurate picture of a student’s overall emotional functioning. Emotional states fluctuate naturally across days and weeks in response to everyday factors such as stress, social interactions, sleep quality, and classroom demands [23,24,25,26,27,28,29,30,31]. For example, Braund and colleagues [31] found that students’ well-being classifications shifted notably when the same survey was repeated six and twelve weeks after baseline, suggesting that single-time-point screening may be unstable for a sizable minority of students. Consequently, a single measurement may provide an incomplete representation of a student’s typical emotional functioning, particularly for those who experience intermittent stress or temporary dips in mood.

Such variability introduces risk into threshold-based screening decisions. When emotional well-being fluctuates around a threshold, a student’s classification may depend on the timing of assessment rather than their broader pattern of functioning. From a practical standpoint, this creates two forms of decision risk. Some students whose emotional well-being is persistently low may not be identified if assessed during a relatively better period, while others whose well-being is generally adequate may be flagged during a transient low. Both scenarios carry real-world consequences: delayed access to support for some students, and unnecessary concern, stigma, or resource use for others [31,32,33,34].

These concerns have also been highlighted in recent work examining stakeholder perspectives on school-based mental health screening, which emphasise sensitivity to misclassification and unintended consequences in school settings [35]. Although some screening frameworks attempt to mitigate these risks through follow-up assessments [22,31], such approaches still rely on an initial classification derived from a single measurement occasion.

### 1.3. The Case for a Mood Monitoring Approach to Screening

In psychological research, repeated assessment of affective states is widely used to examine emotional variability over time. Approaches such as daily diary and ecological momentary assessment (EMA) have shown that emotional experiences comprise both relatively stable components and meaningful short-term fluctuations across days and weeks [36,37]. Capturing this temporal variation provides a richer representation of emotional functioning than single assessments and allows patterns of change and stability to be examined directly.

Although daily diary and EMA methods capture temporal variation in emotional experiences, they are typically used to study emotional dynamics rather than to inform screening decisions in applied educational settings. The present study extends this work by examining how repeated mood monitoring can be implemented within a school-based screening context and how different approaches to aggregating monitoring data may influence identification of students who may require follow-up support. Importantly, this approach is designed to be feasible within routine school practices, relying on brief assessments that can be implemented at low cost and interpreted using simple classification rules.

Mood monitoring represents a potential application of these principles within school settings, involving brief, repeated self-reports of emotional experience over time. Prior research with adults and adolescents suggests that regularly recording emotions is associated with increased affective awareness and reflective engagement, and may prompt greater attention to patterns in emotional experience [38,39,40]. These processes are conceptually aligned with social and emotional learning (SEL) frameworks [41,42], although the present study does not evaluate educational or intervention outcomes.

From a measurement perspective, repeated assessment can improve the interpretability of screening data by integrating information across multiple observations. Aggregating repeated responses reduces the influence of transient fluctuations and allows distinction between short-term variation and more persistent patterns of emotional experience. Importantly, this approach does not redefine the purpose of screening but provides additional temporal context for interpreting threshold-based classifications.

Together, this literature supports examining mood monitoring in school settings from both a measurement and experiential perspective. In addition to evaluating how repeated assessment affects the stability of emotional well-being classifications, it is important to understand how students perceive the monitoring process itself. Examining students’ perceptions of the personal impact of mood monitoring provides important contextual information for interpreting monitoring data and understanding how repeated assessment is experienced within routine school practices. Although the present study focuses on school settings, similar monitoring approaches may also be applicable in other educational contexts, including university populations where emotional well-being concerns are also common.

### 1.4. The Present Study

The present study examined emotional well-being in school students using a repeated mood monitoring approach, with the aim of evaluating the stability of screening classifications across time. Data were collected from two secondary schools, one in the United Kingdom and one in Australia, with students completing weekly assessments of emotional well-being across six to seven weeks.

The primary aim was to examine how often students’ emotional well-being classifications crossed above and below a predefined low well-being threshold across monitoring occasions. This allowed assessment of the extent to which classification status was stable or sensitive to the timing of assessment when emotional well-being was measured repeatedly. Given the descriptive and decision-focused nature of these aims, the study was not designed to test directional hypotheses, but to characterise classification patterns under repeated assessment in real-world school settings.

A second aim was to examine how different decision rules for aggregating repeated monitoring data influence which students are flagged for follow-up. In the present study, we flagged low well-being students to the schools by making use of student averaged scores across their monitoring points in reference to the BEES low well-being threshold value. We contrast this approach with using a student’s first response in reference to the BEES low well-being threshold (i.e., analogous to a single-time-point approach), and with requiring a student to have all monitoring points below threshold (i.e., a more conservative emotional stability-based approach).

The final aim was to examine students’ perceptions of the personal impact of the monitoring process. Specifically, we assessed whether students felt that regular self-assessment supported their understanding of their emotional well-being and whether it related to communication about well-being with others. Together, these aims focus on how emotional well-being classifications change when students are assessed repeatedly in school settings. The study does not aim to diagnose mental health conditions or evaluate interventions, but to understand how repeated measurement influences the interpretation of threshold-based screening decisions.

## 2. Materials and Methods

### 2.1. Study Design

The present study employed a naturalistic, repeated-measures observational design to examine the implications of mood monitoring for school-based mental health screening. The study addressed decision-focused methodological questions concerning (a) the stability of emotional well-being classifications under repeated assessment, and (b) how alternative rules for aggregating monitoring data influence which students are flagged for follow-up. Data collection was embedded within routine school practices to reflect how mood monitoring would be implemented in everyday school settings. No experimental manipulation, randomisation, or intervention was introduced, and all measures were based on student self-report.

Repeated weekly assessments were used to capture within-person variation in emotional well-being across a school term, allowing examination of the stability and fluctuation of students’ well-being classifications relative to a predefined low well-being threshold. Accordingly, analyses focused on classification patterns under repeated assessment rather than on modelling developmental trajectories, identifying predictors of emotional change, or examining underlying causal mechanisms.

The study was descriptive in nature and was not designed to support causal inference, clinical diagnosis, or evaluation of mental health interventions. Emotional well-being thresholds were treated as operational indicators of potential need for follow-up, not as diagnostic determinations. This framing reflects the study’s applied focus on screening practices in school settings [10,11].

### 2.2. Participants

Participants were drawn from two schools that participated in a trial of weekly mood monitoring conducted during a single school term. One school was in the United Kingdom and the other in Australia, providing a cross-national sample drawn from comparable educational contexts. The inclusion of two schools from different national settings was intended to examine mood monitoring under varied but realistic school systems, rather than to conduct direct cross-country comparisons.

The United Kingdom school sample comprised students from Years 7 to 10 (*n* = 413; 45% female) and served a socioeconomically disadvantaged community. The Australian school sample comprised students from Years 6 to 9 (*n* = 354; 54% female) and served a socioeconomically mixed community. Schools were selected in collaboration with educational partners and were typical of mainstream secondary and upper-primary school settings within their respective regions. Although year group labels differ between the two education systems (e.g., UK Year 7 corresponding to Australian Year 6), cohorts were comparable in terms of student age, allowing analyses to be conducted using age-based groupings. Data collection at both schools occurred during the middle of the academic term and did not coincide with major examinations, school events, or excursions.

All students within the relevant year groups at each participating school were invited to participate in the monitoring during scheduled class time. An opt-out consent procedure was used in both schools, consistent with established practices for low-risk school-based mental health research [11,43]. Parents or guardians were informed about the study prior to commencement and could withdraw their child at any time, while student participation remained voluntary. This approach was approved by the relevant institutional ethics committee and implemented alongside standard confidentiality and data protection safeguards.

Based on school-provided estimates of enrolment within the relevant year groups, the analysed sample represented approximately 83% of eligible students in the Australian school and 77% in the United Kingdom school, based on students who completed at least two monitoring assessments during the study period. A minimum of two monitoring assessments was required to ensure responses could be meaningfully linked across time and to reduce the likelihood of including incomplete or misattributed records (e.g., due to inconsistencies in participant codes). Students who completed only a single assessment were therefore excluded from analysis.

Participation varied across monitoring occasions, consistent with typical engagement patterns in opt-out school-based monitoring initiatives. In the United Kingdom school, the proportion of students completing between two and six assessments was 21% (two), 30% (three), 28% (four), 19% (five), and 2% (six). In the Australian school, the proportion completing between two and seven assessments was 17% (two), 17% (three), 17% (four), 22% (five), 17% (six), and 10% (seven).

Ethical approval for the study was obtained from the Edith Cowan University Human Research Ethics Committee (Reference: 2022-03410-ROGERS). Data collection procedures were designed to ensure confidentiality and to minimise any potential risk or burden to students.

### 2.3. Measures

#### 2.3.1. The Brief Emotional Experience Scale (BEES)

Students’ emotional well-being was assessed using the Brief Emotional Experience Scale [44,45]. The BEES is a six-item self-report measure comprising three positively valenced (happy, calm, confident) and three negatively valenced (worried, sad, afraid) emotional adjectives. Each item is rated on a 4-point Likert scale ranging from 0 (not at all) to 3 (a lot), with responses reflecting students’ experiences over the preceding week. These items reflect positive and negative emotional experiences that together provide a brief indicator of overall emotional well-being. Prior validation research has shown that these emotional experiences form a coherent well-being construct suitable for brief monitoring contexts [44,45]. In the present study, the BEES demonstrated acceptable internal consistency across monitoring occasions in both school samples. Cronbach’s alpha values ranged from 0.78 to 0.88 in the Australian school and from 0.73 to 0.78 across the first five monitoring occasions in the UK school. The reliability estimate at Time 6 in the UK sample (α = 0.20) was based on only six cases and was therefore considered unstable and not interpretable.

The BEES was selected due to its brevity, low respondent burden, and suitability for repeated administration in school settings. The measure is designed to capture current emotional experience and is sensitive to short-term fluctuations, while still demonstrating adequate reliability for use in repeated assessment contexts. Unlike longer symptom-focused instruments designed primarily for diagnostic screening, the BEES focuses on emotional experience rather than disorder-specific symptomatology, allowing examination of both relatively stable and short-term variations in emotional well-being over time.

BEES scores are calculated by summing responses to the positively valenced items and subtracting the sum of the negatively valenced items, producing a total score ranging from −9 to +9, with higher scores indicating more positive emotional well-being. For descriptive and screening purposes, scores were categorised into low (less than −1), moderate (−1 to +3), and high (greater than +3) emotional well-being bands, consistent with prior validation work [44]. In that validation research, score bands were derived by examining the distribution of BEES scores across K10 psychological distress categories and supported by receiver operating characteristic (ROC) analyses examining discrimination of elevated distress. In the present study, these thresholds were used as operational indicators to examine classification stability and fluctuation over time, rather than as clinical cut-offs or diagnostic criteria.

A weekly recall period was used to balance sensitivity to emotional change with respondent burden. Shorter recall windows (e.g., daily assessment) may increase burden and reactivity, while longer recall periods may obscure meaningful fluctuations due to recall bias. A weekly timeframe also aligns with school routines, supporting consistent participation across monitoring occasions.

#### 2.3.2. Student Feedback on the Monitoring Experience

To examine students’ perceptions of the mood monitoring process, a brief post-monitoring feedback survey was administered several weeks after completion of the monitoring period. The survey comprised five items designed to provide descriptive insight into students’ experiences of repeated self-assessment.

Students indicated their level of agreement with statements assessing whether participation in monitoring helped them (a) better understand their emotional well-being, and whether it was associated with increased inclination to communicate about well-being with (b) friends, (c) a parent or guardian, (d) teachers, and (e) school counsellors. Responses were recorded on a 5-point Likert scale ranging from 1 (strongly disagree) to 5 (strongly agree). These items were designed to provide contextual information regarding students’ experiences of mood monitoring and were not intended to serve as a validated measure of acceptability, engagement, or intervention impact.

### 2.4. Procedure

Mood monitoring was conducted using a brief online self-report survey administered once per week during a single school term. Surveys were completed during scheduled homeroom periods under teacher supervision. Teachers ensured appropriate conduct during survey completion but did not have access to individual student responses.

Students were instructed to reflect on their experiences over the preceding week when completing each assessment. To accommodate school timetables, survey administration occurred between Wednesday and Friday across classes. Monitoring extended for up to six weeks in the UK school and seven weeks in the Australian school, reflecting site-specific scheduling constraints. Following completion of monitoring, students were invited to complete the feedback survey, which was administered separately and anonymously several weeks later.

### 2.5. Analytic Strategy

The primary analytic focus was to examine the stability of emotional well-being classifications under repeated assessment. For each monitoring occasion, BEES scores were classified relative to the predefined low well-being threshold. Classification patterns were then examined across all available time points for each student. Based on these repeated classifications, students were grouped into descriptive patterns reflecting whether scores remained consistently above threshold, consistently below threshold, or crossed above and below the threshold across monitoring occasions. These patterns were used to characterise classification stability and sensitivity to assessment timing. Students contributed between two and seven monitoring responses.

To examine how different approaches to aggregating monitoring data influenced identification outcomes, classifications based on averaged monitoring scores, single-time-point assessments, and conservative stability-based criteria were compared descriptively. Differences in classification outcomes across approaches were examined using McNemar tests for paired categorical data, and agreement between approaches was assessed using proportion agreement. Gender differences in classification rates were examined using chi-square tests.

To assess whether averaged emotional well-being differed across demographic groups, a factorial analysis of variance (ANOVA) was conducted with year group, school, and gender included as between-subjects factors. This analysis was conducted to identify any large subgroup differences that might warrant stratified reporting of classification outcomes.

All analyses were conducted using IBM SPSS Statistics (Version 30). Analyses were based on all available data for each student, with classification patterns and averaged scores defined according to the number of completed monitoring assessments. Student feedback data were analysed descriptively using frequency distributions and summary statistics. These analyses were intended to provide contextual insight into students’ experiences of mood monitoring rather than to evaluate outcomes or test hypotheses.

## 3. Results

### 3.1. Patterns of Stability and Fluctuation in Emotional Well-Being Classifications

**Figure 1:**
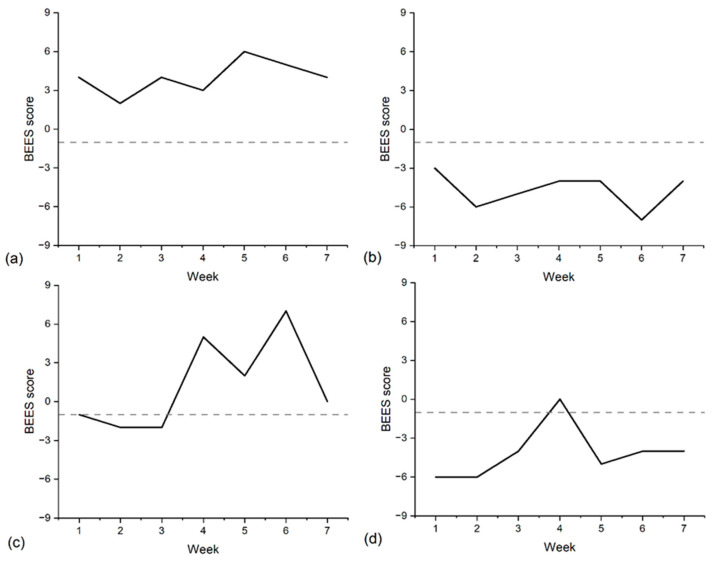
Four examples of student emotional wellbeing scores over seven consecutive weekly check-ins. Solid lines represent participant data, and dashed lines represent the low well-being threshold. (**a**) An example student consistently above the low well-being threshold point across all weeks with 78% of students fitting this pattern. (**b**) An example student consistently below the low well-being threshold point across all weeks with 5% of students fitting this pattern. (**c**,**d**) Examples of students that fluctuated above and below the low wellbeing threshold point with 17% of students fitting this pattern.

Across the monitoring period, three broad patterns of emotional well-being classification were observed when students’ BEES scores were examined relative to the predefined low well-being threshold across repeated weekly assessments. Across all students, the majority (78%) were classified above the low well-being threshold across all monitoring occasions, indicating consistently above-threshold classifications across the available observations. An illustrative example of this pattern is shown in Figure 1a.

A much smaller proportion of students (5%) were classified below the low well-being threshold at all completed monitoring occasions, indicating consistently below-threshold classifications across the monitoring period (Figure 1b). The remaining 17% of students exhibited classifications that crossed above and below the low well-being threshold across monitoring occasions. For these students, classification status varied across weeks rather than remaining consistently above or below the threshold. Illustrative examples of this fluctuating classification pattern are shown in Figure 1c,d.

Students contributed between two and seven monitoring assessments. Classification patterns were defined conditionally on the number of observations available for each student, with no minimum completion threshold imposed to preserve the naturalistic structure of the data. To examine whether classification patterns were influenced by the number of monitoring assessments, we compared students with higher (≥4) versus lower (≤3) numbers of observations. Among students with ≥4 observations, 80% were consistently classified above the low well-being threshold, 4% were consistently below the threshold, and 16% exhibited fluctuating classifications. A similar distribution was observed among students with ≤3 observations (77% consistently above threshold, 5% consistently below threshold, and 18% fluctuating), indicating that classification stability patterns were not meaningfully dependent on the number of monitoring observations.

### 3.2. Flagging Students Under Alternative Classification Approaches

Before examining differences in classification outcomes under alternative classification approaches (summarised in Table 1), we conducted an initial descriptive analysis to assess whether averaged emotional well-being scores differed systematically across schools, year groups, or gender. This analysis was intended to identify any large subgroup differences that might warrant stratified reporting of subsequent classification results, rather than to test substantive hypotheses.

A 4 (year group: ~11–12, ~12–13, ~13–14, ~14–15 years) × 2 (school: Australian, UK) × 2 (gender: female, male) ANOVA was conducted on students’ averaged BEES scores. There was no statistically significant main effect of school, a small effect of year group (F(3,751) = 4.07, *p* = 0.007, partial-η^2^ = 0.02), and a moderate-to-large effect of gender (F(1,751) = 89.87, *p* < 0.001, partial-η^2^ = 0.11), with female students (M = 1.67, SD = 3.11) reporting lower averaged emotional well-being than male students (M = 3.78, SD = 3.03). A small interaction between school and year group was also observed (F(3,751) = 2.98, *p* = 0.03, partial-η^2^ = 0.01). All other interaction effects were non-significant. Given the observed gender differences, classification outcomes are reported separately for female and male students below in Table 1. School and year-group differences were not a primary focus of subsequent analyses, consistent with the descriptive and decision-focused aims of the study.

Classifications based on the first monitoring assessment and classifications based on averaged monitoring scores produced very similar overall rates of low well-being classification. Across the total sample, when collapsing classifications to flagged versus not flagged, agreement between these two approaches was high (92%), indicating that most students received the same classification under both approaches, while a minority were classified differently depending on whether a single time point or averaged monitoring data were used. A McNemar test indicated no significant difference in the overall proportion of students classified as low well-being across the two approaches (χ^2^(1) = 2.02, *p* = 0.16). The same pattern of high agreement with modest disagreement was observed for both female and male students.

In contrast, both first-time-point and averaged-score classifications flagged substantially more students as low well-being than a conservative stability-based criterion requiring all monitoring points to fall below threshold. Compared with this stability-based approach, significantly more students were classified as low well-being when using either the first monitoring assessment (McNemar χ^2^(1) = 66.13, *p* < 0.001) or averaged monitoring scores (McNemar χ^2^(1) = 54.15, *p* < 0.001). This illustrates how different operationalisations of repeated monitoring data can yield materially different identification outcomes despite drawing on the same underlying responses.

Across all classification approaches, female students were significantly more likely than male students to be classified as low well-being. This pattern was observed for classifications based on the first monitoring assessment (χ^2^(1) = 23.70, *p* < 0.001), averaged monitoring scores (χ^2^(1) = 23.85, *p* < 0.001), and the stability-based criterion requiring consistently low scores across all monitoring occasions (χ^2^(1) = 11.10, *p* < 0.001). In all cases, the proportion of students classified as low well-being was higher among females than males.

### 3.3. Student-Reported Experiences of the Mood Monitoring Process

Student feedback was collected to provide contextual information regarding participants’ self-reported experiences of repeated mood monitoring. This feedback was intended to complement the classification analyses and was not designed to evaluate intervention effects. Responses were obtained from 295 students (106 from the Australian school and 189 from the UK school), representing 39% of the monitored sample.

**Figure 2:**
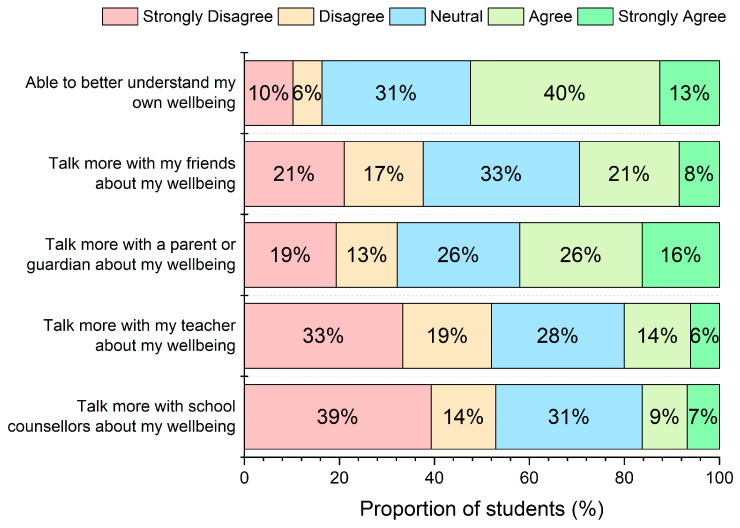
Student level of agreement with questions that aim to assess how the mood monitoring experience impacted on student’s ability to better understand their own wellbeing, and tendencies to communicate with others about their wellbeing.

Overall, 53% of respondents agreed or strongly agreed that participating in mood monitoring supported their understanding of their own emotional well-being (Figure 2). Responses to items assessing communication about emotional well-being varied by social context. Between 29% and 42% of respondents agreed or strongly agreed that participation in monitoring increased their inclination to talk about their well-being with friends or with parents or guardians. Agreement was lower for items referring to communication with school-based adults. Specifically, between 16% and 20% of respondents agreed or strongly agreed that participation in monitoring increased their inclination to talk about their well-being with teachers or school counsellors.

## 4. Discussion

This study examined mood monitoring as an alternative temporal approach to single-time-point mental health screening in schools, with the aim of understanding how repeated assessment influences the interpretation of emotional well-being classifications. The Discussion is structured around three core issues raised by the findings: (a) the extent to which students’ screening classifications remain stable or fluctuate across repeated assessments, (b) how different approaches to aggregating monitoring data influence which students are flagged for follow-up, and (c) students’ self-reported experiences of participating in repeated mood monitoring. Throughout, the findings are interpreted with reference to their implications for screening practice rather than diagnosis or intervention.

### 4.1. Fluctuation in Emotional Well-Being Classifications over Time

This study examined how students’ emotional well-being classifications change when screening is repeated over several weeks rather than conducted at a single time point. Most students (approximately 78%) showed stable classifications above the low well-being threshold across monitoring occasions, while a small proportion (around 5%) were consistently below threshold. Importantly, nearly one in five students (approximately 17%) moved above and below the threshold across weeks, indicating that their classification depended on when assessment occurred. The finding that most students in the present study showed stable levels above the low-wellbeing threshold is consistent with a large body of research on adolescent depressive symptom trajectories, which typically identifies a large low-symptom group alongside smaller groups exhibiting elevated or increasing symptom patterns [46,47,48]. The present study focuses on stability in classification outcomes rather than stability in continuous scores. While indices such as intra-class correlations quantify overall variability across time, they do not capture changes in classification status, which are most relevant in screening contexts where decisions are based on threshold-defined categories.

Single-time-point screening implicitly assumes that emotional well-being is reasonably stable over short periods [12,13,14,15,16,17,18,19,20,21,22]. The present findings indicate that this assumption does not hold for all students. For those whose scores fluctuate around a threshold, a single assessment captures only one moment within a broader pattern of emotional experience. These fluctuations should not be interpreted as measurement error or inconsistent responding. Rather, they are consistent with substantial evidence that emotional experiences vary over relatively short time periods, including week-to-week variation [23,24,25,26,27,28,29,30,31].

From a screening perspective, this variability has important implications. Classifications based on a single assessment may not reflect a student’s typical emotional functioning, particularly for those whose scores fluctuate around a threshold. By contrast, repeated mood monitoring makes it possible to examine classification stability across time, allowing screening results to be interpreted within a broader temporal pattern rather than as isolated observations.

An additional consideration is that fluctuations in emotional well-being classifications may partly reflect contextual influences during the monitoring period, such as everyday academic demands or social experiences. In the present study, monitoring was conducted during the middle of the school term and did not coincide with major examinations or structured school events. This suggests that observed fluctuations are unlikely to be attributable solely to identifiable external stressors, and may instead reflect typical variation in students’ emotional experiences over time. From an applied perspective, this further supports the value of repeated monitoring approaches, as they reduce the risk that classification outcomes are disproportionately influenced by transient, context-specific factors that may distort single time-point assessments.

### 4.2. Flagging Students Under Repeated Mood Monitoring

A central practical question in school-based screening concerns how data from repeated assessments should be used to flag students for follow-up. In a monitoring context, this involves decisions about how multiple observations are combined to determine whether a student meets a predefined screening threshold. In the present study, students were flagged if their average emotional well-being score across the monitoring period fell below the low well-being threshold. This approach was selected for its simplicity and transparency: averaging is straightforward to implement, easy to communicate to school staff, and does not rely on complex decision rules or modelling assumptions.

One limitation of this approach is that flagging occurs only after the monitoring period has concluded. Consequently, students experiencing sustained low well-being may not be identified until several weeks have passed, whereas earlier action might have been possible if decisions were based on initial assessments. From a practical perspective, averaging therefore prioritises certainty over immediacy.

At the same time, averaging across repeated assessments offers an important interpretive advantage. By reducing the influence of isolated low scores, this approach increases confidence that flagged students have experienced persistently lower emotional well-being across the monitoring period, rather than a transient downturn. In the present study, there was high agreement (92%) between classifications based on averaged scores and those derived from a single time point. However, the remaining discrepancy (8%) highlights cases in which classifications differed depending on how information was aggregated. In such cases, decisions based on averaged scores may be more representative of students’ typical emotional functioning, as they incorporate multiple observations rather than a single snapshot.

**Figure 3:**
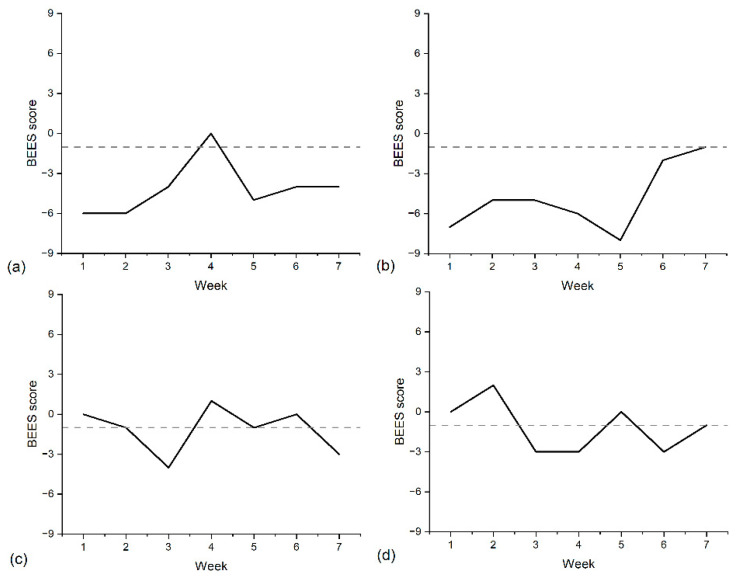
Four examples of students that were flagged as having average BEES score below the low well-being threshold. Solid lines represent participant data, and dashed lines represent the low well-being threshold. (**a**,**b**) Examples of students with consecutive weeks well below the low well-being threshold. (**c**,**d**) Examples of students that despite an averaged flagged score have several weeks above the low well-being threshold.

A further advantage of a monitoring approach is that longitudinal data can be examined alongside threshold-based classifications. For flagged students, patterns of change across time can provide additional context to inform follow-up decisions. Figure 3 illustrates four students whose average scores fell below the threshold. Two examples (Figure 3a,b) show consecutive weeks well below the threshold, suggesting sustained low well-being and a clear case for follow-up. In contrast, the examples in Figure 3c,d fluctuate around the threshold despite an overall average below it, indicating a more ambiguous pattern. In such cases, immediate follow-up may be less clearly warranted, and monitoring patterns may instead be used to guide ongoing observation.

In resource-constrained settings, schools may adopt more conservative criteria for flagging students. One such approach is to flag only students whose scores remain below threshold at all monitoring points. Applying this criterion in the present study resulted in 5% of students being flagged, compared with 12% when using an averaged-score approach. The smaller group identified by this conservative criterion can be conceptualised as students experiencing low well-being with very high confidence, and with a reduced risk of misclassification.

However, a strictly conservative approach also increases the risk of missing students who may benefit from follow-up but whose well-being fluctuates. A potentially more balanced strategy is therefore to use an averaged-score criterion for initial flagging, followed by examination of individual longitudinal patterns to inform decisions about direct follow-up. Such an approach may help schools manage limited resources while reducing the likelihood that students experiencing meaningful distress are overlooked. At the same time, variation in school resources and support capacity means that no single flagging rule is likely to be optimal across all contexts. In settings where resource limitations would otherwise preclude screening altogether, more conservative criteria may be a defensible and pragmatic option.

In addition to differences across classification approaches, gender differences were observed in the likelihood of being flagged for follow-up, with female students more likely than male students to fall below the low well-being threshold across all approaches. This pattern is broadly consistent with existing research indicating that adolescent females are more likely to experience elevated and persistent depressive symptom trajectories [46,47], as well as higher rates of depressive symptoms more generally [49,50,51,52].

The implication for screening is less straightforward. Using a single threshold applies a common standard, but it leads to different rates of identification across groups, as seen here. An alternative is to use subgroup-specific thresholds, such as gender-normed cut-offs, which have been examined in school-based screening contexts as a means of adjusting classification relative to group distributions [53]. In practice, this would reduce the number of female students identified and increase the number of male students flagged. Therefore, the two approaches are not equivalent. A common threshold identifies students below an absolute level of well-being, whereas subgroup-specific thresholds identify those who are low relative to others in their group.

Each approach has consequences. Subgroup-specific thresholds may produce more balanced identification rates, but they may also reduce sensitivity to absolute levels of distress in groups with lower overall well-being. A single threshold avoids this but may result in uneven identification across groups. In the context of school-based screening, there is a strong rationale for prioritising identification based on absolute levels of well-being. Under this approach, students who are flagged represent those with the lowest reported well-being, regardless of group membership, ensuring that follow-up decisions are anchored to level of need rather than relative standing within a subgroup.

### 4.3. Student-Reported Experiences of Mood Monitoring

A mood monitoring approach to screening may also have implications beyond the identification of students for follow-up. In the present study, student feedback was collected to gain insight into students’ self-reported experiences of participating in repeated mood monitoring, with a focus on perceived impacts on emotional understanding and communication about well-being.

Among students who completed the feedback survey, just over half (53%) reported that repeated monitoring supported their understanding of their own emotional well-being. While this finding should be interpreted cautiously, it provides encouraging preliminary evidence that repeated mood monitoring may support students’ emotional awareness. Such effects, if replicated, may have relevance for broader socio-emotional learning initiatives within schools [41,42].

Endorsement was lower for items relating to communication about well-being. Students were more likely to report increased communication with friends or parents or guardians than with teachers or school counsellors. Nonetheless, between 29% and 42% of students indicated that participation in monitoring influenced them to talk more with friends or family about their well-being. This pattern suggests that repeated monitoring may have greater influence on informal help-seeking pathways than on engagement with formal school-based support.

### 4.4. Limitations and Directions for Future Research

Several limitations should be considered when interpreting these findings. First, the study was conducted in two schools within specific national and socioeconomic contexts, which may limit generalisability. Future research should examine repeated screening across a broader range of educational settings and stages to better understand how contextual factors, such as country, school type, educational level, or socioeconomic composition, influence classification patterns. Second, the present study employed a single screening measure, the Brief Emotional Experience Scale (BEES). Whether similar monitoring-based classification patterns would be observed using alternative wellbeing or symptom-focused screening instruments (e.g., affect balance scales or broader mental health screeners) remains an important avenue for future research.

The present study operationalised mood monitoring using weekly reflections on emotional well-being across up to seven consecutive weeks. However, repeated screening can be implemented in multiple ways. For example, alternative approaches might involve daily assessments over shorter periods or different recall windows. Further research is needed to examine how variations in monitoring frequency and time frame influence classification stability, feasibility, and acceptability in school contexts.

We also contrasted three approaches to flagging students for follow-up: classification based on averaged scores across monitoring occasions, a single time point assessment, and a conservative criterion requiring all monitoring points to fall below threshold. These approaches are not exhaustive. Other decision rules, such as requiring two consecutive scores below threshold, may represent viable alternatives. Comparative evaluation of different flagging criteria is needed to inform evidence-based recommendations for best practice.

Student feedback collected in the present study provided preliminary insight into students’ experiences of mood monitoring. However, this feedback was based on a small set of self-report items. More in-depth investigation of student perspectives, as well as examination of the views of other stakeholders such as parents, teachers, and school administrators, represents an important direction for future research. Existing research on stakeholder perspectives has largely focused on single-time-point screening approaches [11,35,54,55,56]. An additional consideration relates to the possibility that repeated self-assessment of emotional experiences could have unintended negative effects for some students. Although existing research suggests that such risks in school-based mental health screening are generally low [31], future research could examine more directly whether repeated mood monitoring influences students’ emotional experiences or help-seeking behaviours.

Finally, practical considerations are critical when evaluating the feasibility of mood monitoring in schools. Barriers to single-time-point screening, including limited resources and a lack of in-house expertise to collect and interpret screening data, have been well documented. A monitoring approach may be viewed as less feasible in some contexts for similar reasons. Our ongoing work is exploring app-based solutions that may help to automate data collection and processing. However, further research is needed to evaluate whether such approaches meaningfully reduce burden and improve uptake in real-world school settings.

From a practical perspective, implementation of a monitoring-based screening approach would likely involve collaboration between school leadership, wellbeing staff, and classroom teachers. For example, teachers could facilitate brief monitoring activities during designated pastoral care or wellbeing periods, while school wellbeing teams review aggregated results and determine whether follow-up support is required. Future research should also examine how monitoring-based approaches could be integrated with intervention pathways, including whether repeated monitoring improves early identification and support compared with traditional single-time-point screening methods.

## 5. Conclusions

This study examined mood monitoring as an alternative temporal approach to single-time-point mental health screening in schools, focusing on how emotional well-being classifications change when assessment is repeated over time. While most students showed stable classifications relative to a low well-being threshold, a substantial minority moved above and below this threshold across weeks, indicating that classification outcomes can depend on when assessment occurs.

Repeated mood monitoring makes this temporal variation visible, allowing screening results to be interpreted in relation to patterns observed across time rather than as isolated observations. In this way, monitoring provides information about the stability of classifications that cannot be inferred from a single assessment. The study conceptualises mood monitoring as a means of informing the interpretation of screening data rather than as a diagnostic or intervention tool. By capturing short-term variation in emotional experience, repeated assessment offers a complementary perspective on emotional well-being under routine school conditions. In addition, preliminary student feedback suggested that repeated mood monitoring was perceived by some students as supporting emotional understanding, indicating that such approaches may be acceptable to students when implemented under routine school conditions.

## Figures and Tables

**Table 1:** The proportion of students classified as low, moderate, and high emotional well-being under three alternative approaches to aggregating monitoring data.

	Averaged Score	First Response Only	All Monitoring Points
Total (n = 767)	L: 12% M: 39% H: 49%	L: 14% M: 40% H: 46%	CB: 5% MX: 17% CA: 78%
Females (n = 378)	L: 18% M: 48% H: 34%	L: 20% M: 48% H: 31%	CB: 7% MX: 24% CA: 69%
Males (n = 389)	L: 6% M: 29% H: 64%	L: 8% M: 32% H: 60%	CB: 2% MX: 11% CA: 87%

L = Low wellbeing, M = Moderate wellbeing, H = High wellbeing, CB = Consistently below low wellbeing threshold, MX = Mixed scores above and below low threshold, CA = Consistently above low wellbeing threshold.

## Data Availability

Data can be found online at: https://doi.org/10.6084/m9.figshare.29874713.v2. Data posted 2 February 2026.

## Abbreviations

The following abbreviation is used in this manuscript:

BEES	Brief Emotional Experience Scale
